# Bladder perforation as a rare complication of urethra sounding with a blunt marking pen

**DOI:** 10.1093/jscr/rjad032

**Published:** 2023-02-07

**Authors:** Mariya Reztsova, Kevin S Chien, Kendall T Post, Alexander Canales, Shinichiro Yokota

**Affiliations:** College of Medicine, Drexel University, Philadelphia, PA, USA; Department of Surgery, Allegheny Health Network, Pittsburgh, PA, USA; Department of Surgery, Allegheny Health Network, Pittsburgh, PA, USA; Department of Surgery, Allegheny Health Network, Pittsburgh, PA, USA; Department of Surgery, Allegheny Health Network, Pittsburgh, PA, USA

**Keywords:** urethral sounding, bladder perforation, laparotomy

## Abstract

Urethral sounding is the insertion of an object or liquid into the urethra for sexual gratification. It is associated with a substantial risk of loss of the foreign body in the bladder, urethral strictures or infection. Bladder perforation is a rare complication of urethral sounding which is usually associated with a sharp object. Here, we present the case of a young adult female presenting with abdominal pain after practicing urethral sounding with a blunt marking pen. She was found to have an intraperitoneal bladder perforation, requiring exploratory laparotomy and bladder repair.

## INTRODUCTION

Urethral sounding is the insertion of an object or liquid into the urethra [1, 2]. The most common reason for deliberate insertion of a foreign body is sexual gratification [3]. However, this relatively uncommon sexual practice of urethral sounding is associated with significant risks of infection, retention of foreign body and urethral stricture [4]. We present a unique case of a female patient who presented after urethral sounding using a blunt marking pen and had intraperitoneal bladder perforation requiring exploratory laparotomy.

## CASE REPORT

A young adult female without significant past medical history presented to emergency department after inserting a pen into her urethra ~12 hours before her arrival while intoxicated. She was unable to retrieve the marker and subsequently developed lower abdominal pain. The patient had some nausea and emesis, which the patient attributed to alcohol.

On presentation, the patient was afebrile, hemodynamically stable with body temperature of 37°C, heart rate of 96 per minute and blood pressure of 151/90 mmHg. Blood work showed leukocytosis of 20.57. Comprehensive metabolic panel was unremarkable (data not shown). Lactic acid was 1.8 mmol/l and was within normal limits. Abdominal examination showed lower abdominal tenderness to palpation but no obvious signs of peritonitis.

**Figure 1:**
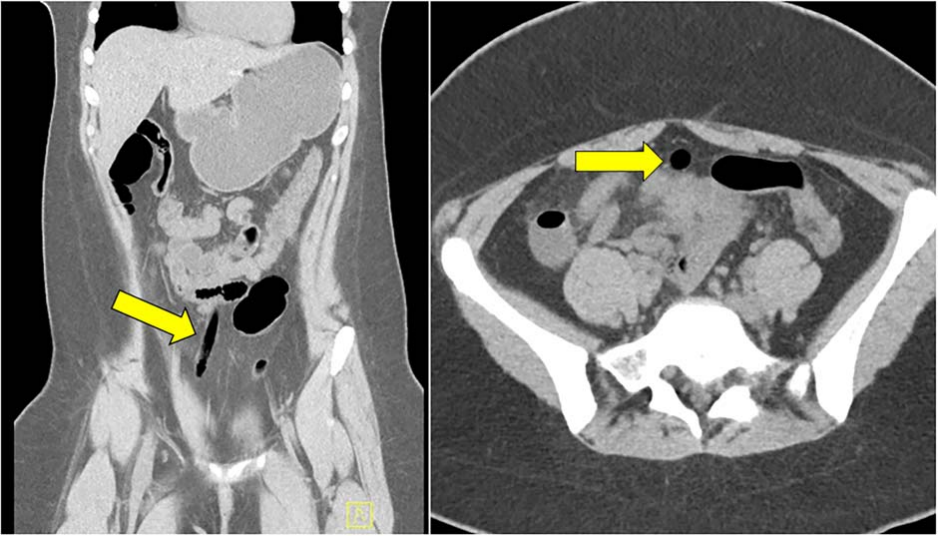
CT scan of abdomen and pelvis showing the presence of an air-filled foreign body in coronal and axial view.

Computed tomography (CT) abdomen and pelvis demonstrated an air-filled tract from the pelvis to mid abdomen, suggestive of a foreign body in the anterior abdomen at the level of the umbilicus (Fig. 1). The radiology report for this CT scan suggested that this foreign body may be located within the urachal remnant.

Urology evaluated the patient and performed a bedside cystoscopy, which demonstrated erythema in the dome of the bladder, but no visible foreign body.

Due to a concern for bladder perforation and a retained foreign body in the abdomen, the patient was urgently taken to the operating room for exploration. It was uncertain whether the foreign body was intraperitoneal or within the urachal remnant. However, we were unable to find the pen with initial exploration using a small midline incision. When we opened the abdominal cavity, we encountered a significant amount of purulent ascites. We extended the midline incision to perform exploratory laparotomy and wash out.

**Figure 2:**
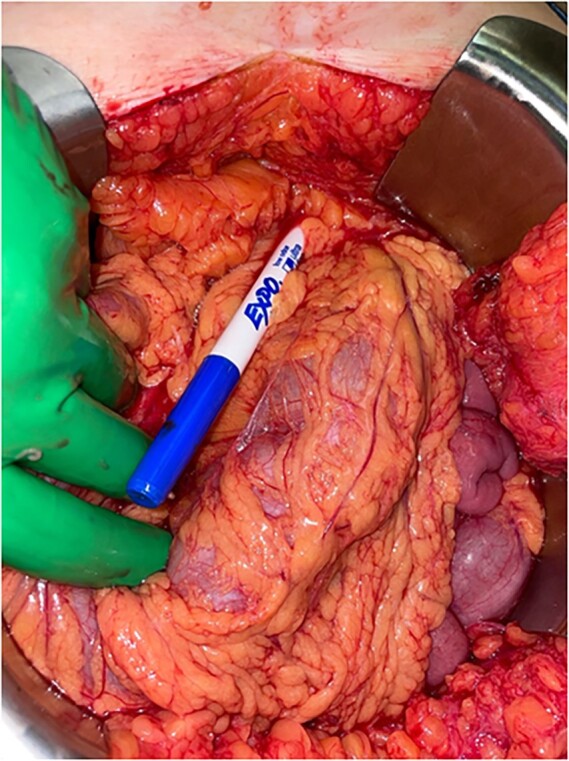
A marking pen was found within the mid-upper abdomen within the greater omentum in parallel to the transverse colon.

**Figure 3:**
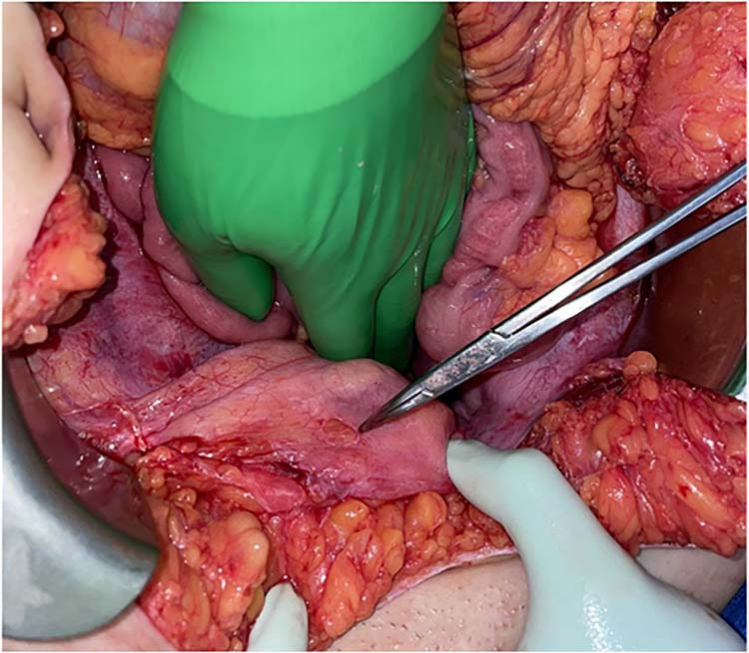
Round defect in the dome of the bladder (pointed with the tip of Tonsil Forceps).

A blunt marking pen was identified in the right upper quadrant, lying on top of the omentum (Fig. 2). Inspection of the bladder revealed a 1-cm round defect in the dome of the bladder (Fig. 3). This was oversewn in two layers, with a subsequent negative leak test. The rest of the abdomen was inspected with no injuries identified to the colon, small bowel, uterus or ovaries. Lastly, the abdomen was washed out and closed. A Foley catheter was left in place and a drain was left by the area of bladder repair.

The post-operative course was unremarkable, and the patient was discharged to home on post-operative day 5. Drain creatinine was tested and its level was consistent with serum creatinine, thus ruling out a concern for urine leak. CT cystogram was obtained before the 2-week follow-up with urology and demonstrated no leak; the Foley catheter was subsequently removed.

## DISCUSSION

Urethral sounding is the practice of inserting objects into the urethra for sexual gratification [1, 2, 4]. A wide variety of objects has been used for this purpose, including metal screws, staples, spoons, thermometers, telephone cables and magnetic beads [5–9].

It is a relatively uncommon sexual practice, and there have been only a few studies that investigated the complications and demographics of those who practice urethral sounding [2, 4, 10]. These studies mainly investigated male population. One study in the USA performed a survey regarding urethral insertion in a population of men with genital piercings, and 385 out of 445 men (24%) admitted to the practice of urethral sounding [10]. Most of these men reported few or only minor complications. Self-reported urinary tract infection (UTI) and urethral irritation were reported by five and four patients, respectively. In another internet-based survey of 2783 men who have sex with men, 228 (10.7%) practiced urethral sounding. Most of the respondent were from North America. The study demonstrated that this group had a significantly higher prevalence of sexually transmitted infections and UTIs/prostatitis, with the odds increasing up to 70% [2].

Only a limited number of reports are available for female patients with urethral sounding [4, 11]. A single institutional case series at a county hospital in the USA described its 15-year experience with urethral foreign bodies. Out of 27 patients, 26 (97%) were male patients and only 1 (3%) was a female patient [6]. In most of these cases, manual extraction with extrinsic pressure, voiding to expel the foreign body and endoscopic retrieval were sufficient for removal. Only one patient required open cystostomy in this case series. Another case series at a hospital in Thailand reported its 20-year experience of 78 female patients with a foreign body in the urinary bladder [8]. A variety of reasons for retained foreign bodies were documented such as illegal abortion, hygienic clean-up, self-dilation for dysuria, iatrogenic migration and sexual abuse/assault. Only 3 out of 78 patients (3.8%) documented masturbation as the reason for insertion, which would be consistent with the practice of urethral sounding. Almost all patients were treated either with cystoscopic removal (44 patients, 56.4%) or cystolitholapaxy (30 patients, 38.4%). This is because of easy access to the bladder via the urethra in female patients [8, 11]. Only four cases (5.1%) required open surgery due to the inability to remove foreign bodies using endoscopic technique. None of these patients had perforated bladder in this case series.

Perforation of the bladder is a rare event after urethral sounding and is usually associated with a sharp object or retained foreign body in the bladder for a prolonged duration [12, 13]. In the present case, the patient presented soon after the insertion of a blunt marking pen. Yet, she suffered from perforation of the bladder, which required exploratory laparotomy and primary repair of the bladder. To our knowledge, this is the first case of intentional self-insertion of a blunt object into the urethra of a female causing an intraperitoneal bladder rupture.

## CONFLICT OF INTEREST STATEMENT

None declared.

## FUNDING

None.

## DATA AVAILABILITY

Data available on request. The data underlying this article will be shared on reasonable request to the corresponding author.
